# Respiratory surveillance for coal mine dust and artificial stone exposed workers in Australia and New Zealand: A position statement from the Thoracic Society of Australia and New Zealand*

**DOI:** 10.1111/resp.13952

**Published:** 2020-10-13

**Authors:** Jennifer L. Perret, Susan Miles, Fraser Brims, Katrina Newbigin, Maggie Davidson, Hubertus Jersmann, Adrienne Edwards, Graeme Zosky, Anthony Frankel, Anthony R. Johnson, Ryan Hoy, David W. Reid, A. William Musk, Michael J. Abramson, Bob Edwards, Robert Cohen, Deborah H. Yates

**Affiliations:** ^1^ Allergy and Lung Health Unit, Centre for Epidemiology and Biostatistics The University of Melbourne Melbourne VIC Australia; ^2^ Department of Medicine Calvary Mater Newcastle Newcastle NSW Australia; ^3^ School of Medicine and Public Health University of Newcastle Newcastle NSW Australia; ^4^ Curtin Medical School Curtin University Perth WA Australia; ^5^ Department of Respiratory Medicine Sir Charles Gairdner Hospital Perth WA Australia; ^6^ Wesley Dust Disease Research Centre Brisbane QLD Australia; ^7^ Health and Management School of Science Western Sydney University Sydney NSW Australia; ^8^ Department of Thoracic Medicine Royal Adelaide Hospital Adelaide SA Australia; ^9^ Christchurch Public Hospital Canterbury District Health Board Christchurch New Zealand; ^10^ Menzies Institute for Medical Research, College of Health and Medicine University of Tasmania Hobart TAS Australia; ^11^ School of Medicine, College of Health and Medicine University of Tasmania Hobart TAS Australia; ^12^ Bankstown Hospital South Western Sydney Local Heath District Sydney NSW Australia; ^13^ Department of Medicine University of New South Wales Sydney NSW Australia; ^14^ Department of Thoracic Medicine Liverpool Hospital Sydney NSW Australia; ^15^ School of Public Health and Preventive Medicine Monash University Melbourne VIC Australia; ^16^ QIMR‐Berghofer Institute of Medical Research Brisbane QLD Australia; ^17^ School of Population Health University of Western Australia Perth WA Australia; ^18^ School of Public Health, University of Illinois Chicago IL USA; ^19^ Department of Thoracic Medicine St Vincent's Hospital Sydney NSW Australia; ^20^ University of NSW Sydney NSW Australia

**Keywords:** coal mine dust lung disease, pneumoconiosis, prevention, respiratory surveillance, silicosis

## Abstract

Coal mine lung dust disease (CMDLD) and artificial stone (AS) silicosis are preventable diseases which have occurred in serious outbreaks in Australia recently. This has prompted a TSANZ review of Australia's approach to respiratory periodic health surveillance. While regulating respirable dust exposure remains the foundation of primary and secondary prevention, identification of workers with early disease assists with control of further exposure, and with the aims of preserving lung function and decreasing respiratory morbidity in those affected. Prompt detection of an abnormality also allows for ongoing respiratory specialist clinical management. This review outlines a medical framework for improvements in respiratory surveillance to detect CMDLD and AS silicosis in Australia. This includes appropriate referral, improved data collection and interpretation, enhanced surveillance, the establishment of a nationwide Occupational Lung Disease Registry and an independent advisory group. These measures are designed to improve health outcomes for workers in the coal mining, AS and other dust‐exposed and mining industries.

**Abbreviations**
RANZCR: Royal Australian and New Zealand College of Radiologists
AS: artificial stone
CMDLD: coal mine dust lung disease
CT: computed tomography
CWP: coal workers' pneumoconiosis
CXR: chest X‐ray
DLCO: diffusing capacity of the lung for carbon monoxide
FEV_1_: forced expiratory volume in 1thinspaces
GLI: Global Lung Function Initiative
ICOERD: International Classification of Occupational and Environmental Respiratory Disease
ILO: International Labour Office
LDCT: low‐dose CT
MCTD: mixed connective tissue disease
MDP: mixed dust pneumoconiosis
MDT: multidisciplinary team
NZ: New Zealand
PMF: progressive massive fibrosis
PPE: personal protection equipment
RCS: respirable crystalline silica
RPP: rapidly progressive pneumoconiosis
TSANZ: Thoracic Society of Australia and New Zealand
uLDCT: ultralow‐dose CT

## INTRODUCTION

The recent reappearance of coal workers' pneumoconiosis (CWP)^1^, ^2^ and emergence of artificial stone (AS)‐associated silicosis^3^, ^4^, ^5^ has represented a failure of preventive systems to protect the respiratory health of workers in Australia. This resurgence of pneumoconiosis has occurred at a time when production has increased, mining techniques have been further mechanized and responsibilities for medical care outsourced, despite state‐ and nation‐specific regulations to control respirable dust levels. In 2016, the Thoracic Society of Australia and New Zealand (TSANZ) highlighted key issues relating to the pressing need to improve periodic health surveillance for coal mine workers.^6^ Some but not all of these recommendations have been implemented.

This paper provides a framework for an optimal surveillance system of these workers in Australia and New Zealand and for improvements in the existing respiratory surveillance programme (Box 1). We emphasize that respiratory surveillance differs from targeted case finding such as has been implemented for AS workers in some states, and also from population screening for non‐occupational diseases (Box 2).^7^

BOX 1 **Goals and directions proposed by the Thoracic Society of Australia and New Zealand**

**Table:** 

**Goals of this review** To promote awareness among physicians and healthcare professionals about artificial stone (AS) silicosis and the spectrum of coal mine dust lung disease (CMDLD), and provide the rationale to support enhanced respiratory surveillance of exposed workers in Australia and New Zealand. To highlight optimal surveillance strategies which will identify workers at risk of CMDLD and AS‐associated silicosis at an early stage in order to allow timely implementation of effective interventions and advance personalized management. **Proposed directions** Improvements in collection of periodic health surveillance data and its interpretation in accordance with established practice and international guidelines, emphasizing longitudinal comparisons within individuals and enabling these data to become available for research and clinical decision‐making. Enhanced surveillance methods to include diffusing capacity measurements, computed tomography for coal miners and AS workers where applicable at as low dose as possible; increased frequency of surveillance for symptomatic and high‐risk groups, and evaluation of these strategies within prospective studies. Improved availability of cumulative dust exposure measurements for supervising medical advisors at the time of examining exposed workers, for respiratory physicians after referral, and to enable improved research into dose–response relationships between exposure and disease. Diagnoses of CMDLD/AS silicosis to be confirmed by occupational respiratory multidisciplinary teams with the recording of all such diagnoses within a centralized national registry as notifiable conditions, facilitated by the establishment of a central expert Occupational Lung Disease Advisory Group.

BOX 2 **Occupational definitions as adapted from Wagner^7^**

**Table:** 

**Respiratory surveillance** Respiratory surveillance is a component of public health practice involving the periodic collection, analysis and reporting of information in a workplace for disease detection and prevention. In contrast to population screening, respiratory surveillance is directed towards improvement of the health of workers who are exposed to a known workplace respiratory risk factor. **Case‐finding of an individual after a ‘sentinel event’ has occurred** Case‐finding uses medical testing to make a presumptive diagnosis of disease before an individual would normally seek medical care, usually when an available intervention can favourably affect the person's health. Case‐finding aims to detect disease in its ‘preclinical’ stage. This allows monitoring of outbreaks and subsequent secondary prevention of disease in workplaces and communities. **Population screening** Screening is defined by the International Labour Office (ILO) as ‘the presumptive identification of unrecognized disease in an apparently healthy, asymptomatic population by means of tests, examinations or other procedures that can be applied rapidly and easily to the target population. A screening programme must include all the core components in the screening process from inviting the target population to accessing effective treatment for individuals diagnosed with disease’. Population screening is not the same as respiratory surveillance.

Detection of coal mine dust lung diseases (CMDLD) and AS silicosis at an early stage should facilitate more effective management and allow avoidance of further dust exposure to benefit individual workers. Our framework is based on existing literature including the review of the Queensland Coal Mine Worker's Health Scheme,^8^ WorkCover Queensland's (WCQ) review of stonemasons with silicosis,^9^ targeted literature searches and international guidelines. It also includes opinion from national and international experts in the field. This framework could potentially be used in construction, crafts and related trades as well as other mining operations which involve exposure to mineral dusts. Although the outbreak of pneumoconiosis has to date primarily affected Australia, this position paper has been developed co‐operatively and includes expertise from both countries, being intended to apply to both Australia and New Zealand. We aim to inform occupational physicians, general practitioners with special training in occupational disease and respiratory surveillance, and to provide a reference framework for respiratory physicians who are referred such patients. We also aim to benefit the workforce more broadly through identifying workplaces and work practices that need further hazard assessment.

This framework will expire 5 years from the date of publication but will be discussed at future TSANZ meetings as per TSANZ standard procedure for position papers. Dust control is critical to disease prevention but is outside the scope of this document. Specific recommendations for dust control are available.^10^, ^11^, ^12^, ^13^

## POSITION STATEMENT

### Clinical aspects of CMDLD

**Figure 1:**
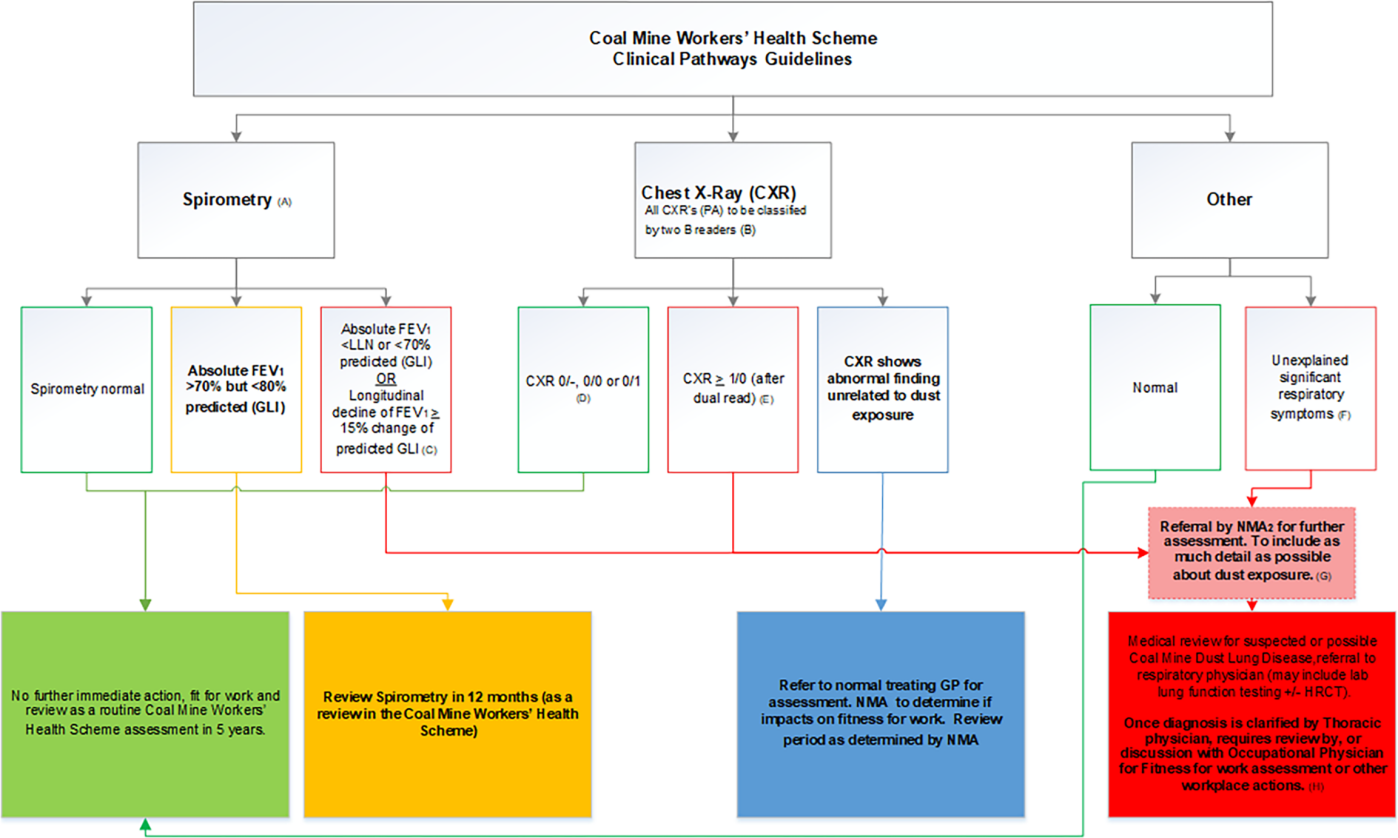
Coal Mine Workers' Health Scheme. This clinical pathway guideline documents the currently recommended process for follow‐up investigation and referral to appropriate medical specialists of workers with abnormal results. Additional information supporting the guideline is available from https://www.dnrme.qld.gov.au/__data/assets/pdf_file/0005/1278563/cmwhs‐clinical‐pathways‐guideline.pdf. Adapted from *Department of Natural Resources, Mines and Energy* of the Queensland Government, with permission. FEV_1_, forced expiratory volume in 1 s; GLI, Global Lung Function Initiative; GP, general practitioner; HRCT, high‐resolution computed tomography; LLN, lower limit of normal; NMA, nominated or supervising medical practitioners; PA, postero‐anterior.

CMDLD comprise several diseases in addition to classical CWP,^1^, ^14^, ^15^ an interstitial lung disease resulting from chronic inhalation of coal mine dust, otherwise known as ‘black lung’. CWP is characterized by nodular, and less commonly irregular opacities, on plain chest radiographs.^1^ Most coalmine dust also contains a component of respirable crystalline silica (RCS) which is more fibrogenic than coal dust, potentially leading to silicosis or mixed dust pneumoconiosis (MDP).^16^ The spectrum of CMDLD, which includes chronic obstructive pulmonary disease (COPD), is summarized in Table 1.^15^, ^17^ Notably, diagnoses can be missed by medical surveillance programmes that focus only on classical radiographic‐nodular CWP. The current clinical pathway for CMDLD in Queensland is outlined in Figure 1, where such respiratory surveillance has recently been made mandatory with centralized reporting.

**Table 1:** Spectrum of disease in CMDLD and silicosis

Diagnoses
Bronchial anthracosis^†^ Chronic bronchitis Caplan's syndrome (rheumatoid pneumoconiosis)
Coal workers' pneumoconiosis (CWP) Simple CWP Rapidly progressive pneumoconiosis Progressive massive fibrosis Dust‐related diffuse fibrosis Mixed dust pneumoconiosis Silicosis Acute Accelerated (e.g. artificial stone workers) Chronic (or classical)
COPD (with or without smoking)
Tuberculosis (including latent tuberculosis and non‐tuberculous mycobacterial infection) Lung cancer Kidney disease Autoimmune disorders including MCTD, Sjögren's syndrome and others

Adapted from Perret *et al.*,^15^ with permission.

† Bronchoscopic and/or pathological diagnosis.

CMDLD, coal mine dust lung disease; COPD, chronic obstructive pulmonary disease; CWP, coal workers' pneumoconiosis; MCTD, mixed connective tissue disease.

In individuals exposed to coal mine dusts, CWP may develop into progressive massive fibrosis (PMF), which may ultimately be fatal. PMF may also occur in open cut or surface miners involved in drilling/blasting without a prior history of underground mining.^18^ Rapidly progressive pneumoconiosis (RPP) is defined by the development of PMF and/or an increase in small opacity profusion greater than one International Labour Office (ILO) subcategory over 5 years.^19^, ^20^ This is associated with respiratory failure, right heart failure and premature death. RPP is associated with annual lung function declines of forced expiratory volume in 1 s (FEV_1_) >60 mL/year compared with miners without CWP,^21^ and occurs in CWP, silicosis and MDP. There has been an alarming rise of RPP/PMF diagnosed in the USA over the last 15 years,^1^, ^19^, ^22^, ^23^; it is not yet clear whether this has also occurred in our countries.

CMDLD also includes chronic bronchitis and emphysema, which were documented in coal mine workers in the 1940s.^24^ While initially attributed to smoking alone, coal dust‐related obstructive chronic bronchitis (OCB), emphysema and accelerated lung function decline have since been amply confirmed.^25^, ^26^ These effects are additive rather than attributable to smoking alone and can also occur without radiological CWP on chest radiographs.^25^, ^27^, ^28^, ^29^ Interstitial pulmonary fibrosis related to coal mine dust exposure (dust‐related diffuse fibrosis or DDF) also occurs, and can be identical clinically to other types of progressive fibrosing interstitial lung disease.^1^

### Clinical aspects of AS silicosis

AS silicosis, which progresses more rapidly than classical silicosis, is an emerging global public health issue in workers in the kitchen/bathroom bench‐top industry.^3^, ^5^ Cutting and grinding AS without dust controls such as water suppression and local exhaust ventilation (LEV) can generate very high levels of RCS and also other substances potentially toxic to the lung. Unfortunately, poor work practices have occurred in this industry and periodic surveillance has been infrequently performed.

This progressive form of silicosis is not yet fully understood. Disease may occur within 5 years of exposure, with rapid lung function loss and radiological progression.^4^ Symptoms may be delayed, resulting in severe disease at first presentation.^17^ The clinical features of AS silicosis include lung function restriction, interstitial radiographic nodules and positive auto‐antibodies, similar to those seen in classical silicosis, and younger men are often affected.^3^, ^17^ Shorter latency periods, rapid progression to PMF and silico‐proteinosis‐like patterns on computed tomography (CT) scanning and histopathology may be found.

Lack of any effective treatment has added urgency to primary prevention. Dust suppression, including a combination of wet cutting methods and ventilatory controls, can reduce RCS levels,^30^ but even wet cutting of AS can produce levels that exceed the current workplace exposure standard for Safe Work Australia's time weighted average (TWA) of 0.05 mg/m^3^, which is currently identical in New Zealand (NZ).^11^, ^13^ (see also http://hcis.safeworkaustralia.gov.au/ExposureStandards/Details?exposureStandardID=1042).

Further controls need to be considered in relation to the hierarchy of control, ordered from most to least effective (i.e. product substitution, engineering changes, administrative controls and then personal protection equipment (PPE)), as captured in the Australian and New Zealand Work Health and Safety Legislation: clause 36 of the model Work Health and Safety (WHS) for Australia^11^ and clause 6 of the NZ Health and Safety at Work (general risk and workplace management) Regulation.^13^

Use of PPE is the least effective control measure and therefore should be regarded as the very last resort. Periodic surveillance of workers to detect early disease and optimal case management of diagnosed disease in these young‐to‐middle‐aged workers is critical.^17^ In a recent retrospective review of 78 AS masons in Queensland who had been given a clinical diagnosis of silicosis following active case finding and using high‐resolution CT (HRCT) scanning, 34 (43%) had normal plain chest radiographs.^9^ Of these, at least two‐thirds had spent >50% of their total work tenure cutting and grinding without appropriate dust controls. Some false‐negative findings occurred using chest radiographs alone, and HRCT was a more sensitive diagnostic test.^31^ Although these are early data,^9^ surveillance of workers with high levels of exposure using low‐dose CT (LDCT) is much more likely to detect disease at an earlier stage compared with plain radiographs, when removal from dust exposure can assist long‐term outcomes.

### Improvements in lung function and imaging techniques

Techniques for investigating occupational lung disorders have improved significantly over recent decades and should be incorporated into modern respiratory surveillance methods. Improvements in lung function testing, in predicted values for interpretation, and computerized analysis of results should assist diagnosis. Lung function testing beyond spirometry which includes static lung volumes and diffusing capacity of the lung for carbon monoxide (DLCO),^32^, ^33^, ^34^ should enable detection of the full spectrum of CMDLD. Although DLCO can be reduced by factors including smoking,^35^, ^36^ its ability to evaluate the gas exchange surface is highly relevant for early detection of silicosis and coal‐related emphysema. Furthermore, the equipment has become increasingly portable, so it can easily be taken to the point of testing (e.g. remote mine sites). Other developments include the derivation of internationally accepted reference values,^37^, ^38^ novel software to assist with monitoring rates of longitudinal lung function decline^39^ and the option of secure, cloud‐based centralized data storage.

Importantly, more sensitive and specific radiological techniques have been developed for the detection of lung disease at an earlier stage. The diagnostic utility of HRCT scans is superior to plain chest imaging in both detecting and evaluating small parenchymal opacities and in staging disease in CWP^40^, ^41^, ^42^ and silicosis. Application of computerized software is increasingly allowing quantification of extent of emphysema and interstitial lung disease, as well as assessment of the airway components of COPD. The use of LDCT with typical radiation exposure of 1.5 mSv has also enabled early detection of lung cancers and screening is recommended for those at increased risk in several countries.^43^, ^44^ The ionizing radiation doses of ultralow‐dose CT (uLDCT) protocols now approaches that of plain chest radiographs (0.12–0.20 vs 0.10 mSv),^45^, ^46^ without substantially losing diagnostic quality.^45^, ^47^ This contrasts with the estimated annual exposure for an average Australian of 1.5 mSv from natural sources.^48^ However, uLDCT has not yet been validated for the surveillance of coal‐ and silica‐related pneumoconioses.

CT scans should be interpreted by an experienced thoracic radiologist with understanding of the International Classification of Occupational and Environmental Respiratory Diseases (ICOERD) system^49^ and RANZCR recommendations.^50^ Ideally, the ICOERD system should be updated and adopted internationally. Radiological grading scales provide prognostic risk of disease progression in individuals with advanced disease. For example, coal mine workers with ILO grade >2/1 have an estimated 1 in 8 chance (12.5% risk) of developing PMF within 5 years.^51^ At a population level, such reporting in accordance with occupational imaging classification standards can more accurately estimate incidence, prevalence and severity of occupationally acquired lung diseases.

There is a need to regularly monitor clinician competency in interpreting images, such as the ‘B’ reader course, which has recently been introduced in Queensland, although no such certification exists for CT scanning. TSANZ's course on occupational lung diseases provides the opportunity for an annual update for clinicians, but optimally this area requires further development.

### The way forward: optimizing respiratory periodic health surveillance

#### Improving collection of exposure data (dust sampling)

Exposure to respirable coal mine dust has been subject to much research; however, this is not true for AS exposure. Measurements of workplace RCS are needed to understand cumulative exposures a worker may have experienced but need to be analysed by an accredited analytical facility. Dust monitoring should occur under typical working conditions, not when work is performed at less than usual capacity. Careful review of compliance to national standards for RCS collection and analysis^52^ is required. Ideally, individual exposure data should be available at the time of surveillance, accessible via a centralized database. Although outside the scope of this paper, improved national and international collaboration is needed between occupational health staff, engineers involved in dust control and treating doctors.

A detailed occupational exposure history is essential for all workers including a full chronological history of all jobs, use (or lack of) of appropriate dust controls in current and previous employment and other exposures, particularly concurrent smoking and vaping. Accurate dust measurements suitable for pooled data analysis are required to better inform policy on appropriate respirable dust limits; this requires close collaboration with occupational hygienists.

### Improving health record documentation and interpretation

The review of the coal miners' periodic health surveillance system in Queensland highlighted multiple areas where improvements were needed.^8^ These included poor performance and interpretation of spirometry, and deficiencies in quality control and administrative procedures. Chest radiograph interpretation was limited by suboptimal film quality, lack of clinical information and lack of recognition of early disease. Surface workers exposed to dust in open cut mines were not routinely included in surveillance despite being at risk of CMDLD.^8^ There was lack of a quality assurance programme and of adherence to international standards. The B reader programme using chest radiographs which is used in the USA and is evidence based and relies on regular clinician training^53^, ^54^, ^55^ was not in operation, and no quality control programme for clinician competency was followed.

Our proposed improvements in periodic surveillance programmes for the conduct and interpretation of testing as well as other directions are shown in Table 2. Illustrative references have been provided as a full discussion is beyond the scope of this paper.

**Table 2:** Optimizing periodic health surveillance in the coal and AS industry

**Proposed improvements to periodic health surveillance**	Citation/s
Regular training of staff in accordance with international standards including quality control and quality assurance, including accurate interpretation of lung function data by supervising medical practitioners	8, 32, 33, 39
Plain chest radiographs to be performed using ILO recommended techniques and to be technically acceptable. Classification only by qualified thoracic radiologists, preferably with B‐reader qualifications, and compared with previous images	8, 20
Individual spirometry results to be interpreted using reference values of the GLI, with serial data compared with longitudinal predicted values, while adopting the lower limit of normal to define lung function abnormality	8, 37, 38, 39
Dust monitoring information under typical working conditions (≥75% capacity) and considered if substantive changes are made to facility, processes and practices, to be recorded using an accredited facility, with individualized data available at the time of periodic surveillance	3, 10, 11
Extending surveillance methods for AS exposure to potentially include LDCT, reported by expert thoracic radiologists; careful evaluation of the role for uLDCT for coal miner and AS workers within longitudinal prospective studies	9, 56
Extending surveillance methods for all workers to include lung diffusing capacity according to ATS/ERS standards at intervals of 3 years or less with careful evaluation of such surveillance within longitudinal prospective studies	27, 32, 33, 56
A flexible approach to the timing of surveillance of coal mine dust workers, including annual spirometry and DLCO if results are abnormal but do not yet fulfil diagnostic criteria for disease	7, 56, 57
For AS workers previously exposed to high RCS levels, active case‐finding using conventional HRCT/spirometry/DLCO performed at accredited respiratory laboratories and radiological facilities using recommended protocols; follow‐up by expert treating specialists/teams, preferably at occupational respiratory MDTs	9, 32, 33, 50, 58, 59
For AS workers, pre‐employment plain chest radiographs to exclude major abnormalities. For AS workers undergoing active case‐finding without abnormal CXR or HRCT, annual spirometry/DLCO and imaging 3‐yearly or more often depending on individual factors and test results	17
Complementing surveillance CXR imaging with HRCT scans in high‐risk groups, for example, where borderline fibrosis is found on plain chest radiographs and/or where discrepancy exists with lung function findings	49, 60
Improving existing medical databases to allow capacity to compare serial lung function data, occupational exposure history, imaging findings and dust measurements	39, 61
Early review of the diagnostic utility of ‘best available tests’ (LDCT, uLDCT and DLCO) by comparisons with plain chest radiographs and spirometry collected prospectively with consent from workers, ideally in a research setting	

AS, artificial stone; ATS/ERS, American Thoracic Society/European Respiratory Society; CXR, chest X‐ray; DLCO, diffusing capacity of the lung for carbon monoxide; GLI, Global Lung Function Initiative; HRCT, high‐resolution computed tomography; ILO, International Labour Office; LDCT, low‐dose CT; MDT, multidisciplinary team; RCS, respirable crystalline silica; uLDCT, ultralow‐dose CT.

For surveillance for CMDLD, we recommend that the diagnostic utility of uLDCT needs to be compared with that of the ILO classifications of plain chest radiographs in prospective studies. While randomized controlled trials would provide the best evidence for the evaluation of comparative efficacy, such studies would need to be performed over long periods, due to the prolonged latency periods of these diseases.

When interpreting spirometry, a fixed cut‐off value of post‐bronchodilator FEV_1_/forced vital capacity (FVC) <0.70 has previously been recommended to define airways obstruction.^62^ However, this under‐diagnoses airflow obstruction for younger and taller working adults up to their early 40s, and overestimates this in older, shorter subjects. The internationally recognized Global Lung Function Initiative (GLI) predicted values has allowed better definition of the limits of normality,^37^, ^38^ and should be adopted; multiple serial measurements are needed for optimal accuracy.^39^

Databases used for health surveillance need to compare serial lung function data over a working life. It is highly desirable to link these databases with occupational histories and occupational hygiene data, enabling medical practitioners to be better informed regarding type, duration and intensity of exposures. Doctors assessing serial lung function data require appropriate and ongoing training to identify lung function decline beyond that of age‐related decline and measurement variability, using longitudinal analysis software such as SPIROLA.^39^ The SPIROLA software also allows monitoring of the quality of spirometry for the purposes of quality assurance which is also important for medical surveillance.

### Frequency of surveillance and individual management

Most existing surveillance programmes in the mining industries use a uniform system regardless of individual risks; however, the field is now moving beyond the ‘one‐size‐fits‐all’ approach. Although workers who have been consistently employed in roles associated with higher dust exposures appear at greater risk of developing lung disease, other factors may contribute (e.g. genetic predisposition,^15^ concomitant smoking and/or pre‐existing respiratory diseases). One important benefit from this ‘personalized’ approach could be to facilitate a worker's knowledge of potential adverse effects from respirable dust exposures, provide smoking cessation advice and encourage the reporting of new respiratory symptoms that might represent early disease or disease progression.

The frequency with which respiratory surveillance is performed in coal miners is not standardized within Australia and New Zealand, but it usually occurs 3–5 yearly. International recommendations suggest more frequent lung function testing to identify accelerated lung function decline at an earlier stage.^1^, ^57^ The U.S. National Institute for Occupational Safety and Health (NIOSH) programme consists of mandatory spirometry/chest X‐ray examination upon entry into mining and after 3 years, then annual spirometry and chest X‐ray examination every 2–5 years for both underground and surface miners working in the industry over their lifetime.^7^, ^56^ Findings from mining regions that exceed current respirable dust exposure limits support at least annual testing of spirometry, especially for young smoking miners with bronchitis symptoms,^26^, ^63^ but ideally spirometry should not be dependent on dust measurements. Assessment of cross‐sectional data other than the baseline testing is generally not as useful as serial measurements, especially if a worker begins his/her career with above average readings. Whenever possible, longitudinal patterns of lung function decline should be estimated using the free SPIROLA software.^39^, ^61^ A web‐based version of this software is due for release soon (https://www.cdc.gov/niosh/topics/spirometry/spirola-software.html).

Where radiological and physiological changes are found but do not yet meet diagnostic criteria for clinical disease, more frequent monitoring is indicated on an individual basis, depending on the risk profile. For example, a repeat chest X‐ray in 2 years is suggested for coal mine workers if there is radiological evidence of category 1/0 or higher pneumoconiosis, and annual spirometry if results are below the lower limit of predicted normal and/or if there is accelerated FEV_1_ decline.^56^ Individuals with acute intense exposures may warrant monitoring more frequently. It is important that health surveillance continues after ceasing employment because workers are still at risk in subsequent years,^64^, ^65^ especially if a resignation was prompted by health concerns.^28^ Specialist medical evaluation is appropriate if there is uncertainty.

### Implementing surveillance for AS workers

Given the exceptionally high exposure to RCS in the AS industry in Australia, respiratory surveillance needs to be considered separately from coal mine dust exposure. Prior to the first Australian report of AS silicosis cases,^4^ there was little workplace awareness of existing regulations or the need for appropriate surveillance. Subsequently, specific case finding programmes have been implemented in some states to detect AS silicosis in its ‘preclinical stage’ using conventional HRCT.^50^ Early results suggest that individual cases identified by targeted screening may require frequent monitoring with full lung function tests and CT, given the potential for rapid progression of disease.^4^

The TSANZ supports optimal respiratory surveillance methods in order to increase the detection of early disease. For individuals with significant exposure but no current evidence of disease, there is consensus among professional bodies including TSANZ, RANZCR, Royal Australasian College of Physicians (RACP) and Australasian Faculty of Occupational and Environmental Medicine (AFOEM) that surveillance with LDCT is preferable to plain chest radiography. Currently, it remains unclear whether LDCT is non‐inferior to HRCT. Similarly, annual lung function testing and radiological surveillance at intervals 3‐yearly or less is preferred. Interval cases may occur if the surveillance tests are spaced too far apart. For workers new to the industry, it is not yet clear whether LDCT is feasible as a baseline pre‐employment test.

We acknowledge the lack of evidence regarding enhanced radiological surveillance, and therefore suggest further research. In the interim, RANZCR recommendations should be followed. While we have suggested it may be reasonable to initially adopt LDCT given its use in lung cancer screening, it is essential that future studies fully address the question of whether the sensitivity of LDCT is sufficient to adequately detect smaller and/or ground‐glass nodules that are characteristic of silicosis. A role for uLDCT in occupational respiratory surveillance needs investigation if the use of LDCT becomes established. It is important to recognize that improvements in dust control measures in this industry would be expected to reduce the incidence of AS silicosis, but that lifelong respiratory surveillance will still need to be conducted due to the long latency between exposure and disease development. Respiratory surveillance, as a secondary screening tool, should be conducted regardless of how well dust control measures improve, and should continue after the worker has left the employment and as long as the worker agrees to this. Optimal respiratory surveillance will ensure that new exposures or failures in exposure controls are identified quickly and that remediation occurs, while protecting workers' respiratory health.

### Formalizing clinical pathways to specialist review

For equivocal and confirmed diagnoses identified by case finding or periodic surveillance, early specialist referral to an expert respiratory physician is recommended, while acknowledging potential difficulties with accessibility and costs. These factors are influenced by state, federal and healthcare systems. Establishing links between employers, local, national and international professional bodies can facilitate these processes. As already demonstrated for lung cancer and idiopathic pulmonary fibrosis, diagnosis and recommendations for clinical care are enhanced by a specific multidisciplinary team (MDT) meeting that enables consensus‐based diagnosis and management. Further investigation may require invasive procedures such as bronchoscopy, endoscopic bronchial ultrasound‐guided or surgical lung biopsy. However, it should be emphasized that these procedures are invasive and should only be necessary in few cases. Optimal management includes smoking cessation interventions, psychological counselling and access to supportive care such as pulmonary rehabilitation, vaccination and oxygen therapy where appropriate.^1^ Vigilance is needed given the predisposition to mycobacterial infection after silica exposure. Referral for lung transplantation may be required.^1^ This multidisciplinary approach is likely to improve diagnostic accuracy and improve workers' health.

### Establishing a centralized registry and Occupational Lung Disease Advisory Group

Currently, information regarding the number and type of cases of occupational lung diseases in Australia and New Zealand is very poorly documented. The TSANZ, with the support of key medical professional bodies, has proposed the establishment of a national registry to record all cases of occupationally acquired lung disease. Formal registration of individuals with confirmed occupationally related respiratory diagnoses within this central registry would facilitate gathering of accurate data and allow targeted interventions to protect other workers. Diagnostic accuracy is paramount and should be overseen by experts in occupational lung disease at occupational lung MDT meetings (Occ‐L‐MDT). A system of MDTs is already established in each Australian state and in New Zealand for lung cancer and interstitial lung diseases, and could easily be implemented more widely. The formation of a national advisory group (potentially also to include New Zealand) consisting of expert practitioners in occupational and respiratory medicine, occupational hygiene, radiology and respiratory physiology could provide expert and timely advice to practitioners and governments. Systems for notification of occupational lung diseases are already being reviewed at state and national levels in Australia and New Zealand. The recording of radiological images, lung function measurements and dust exposure data from industry and/or state mine regulatory authorities within a central registry would allow analysis of disease features and monitoring of epidemiological trends nationally.

## CONCLUSIONS

The recent resurgence of CMDLD and the rapid emergence of AS‐associated silicosis in Australia have highlighted a failure of enforcement of current regulations for dust control as well as poor periodic respiratory surveillance. Given these past deficiencies, the TSANZ now provides a framework to enhance existing programmes and comply with international standards. These includes the conduct, interpretation and audit of respiratory surveillance testing, improvements in database systems, a flexible approach to enhance individual surveillance and expansion of surveillance testing to include lung diffusing capacity and CT scanning. TSANZ also recognizes the need to establish a firm evidence base for enhanced surveillance by performing appropriate research. Furthermore, TSANZ proposes the establishment of a national registry for occupationally acquired lung diseases and an independent Occupational Lung Disease Advisory Group. This would facilitate the continued improvement in existing programmes, reduce preventable respiratory disease and promote a positive and supportive workplace culture for Australian and New Zealand workers.

## Disclosure statement

F.B. has received unrestricted research grants to examine optimal radiology approaches for screening AS workers: iCare DDB and Royal Perth Hospital. D.H.Y. has been a PI on a peer‐reviewed grant from the Coal Services Trust, NSW.
